# Ideal Theory in Semigroups Based on Intersectional Soft Sets

**DOI:** 10.1155/2014/136424

**Published:** 2014-06-23

**Authors:** Seok Zun Song, Hee Sik Kim, Young Bae Jun

**Affiliations:** ^1^Department of Mathematics, Jeju National University, Jeju 690-756, Republic of Korea; ^2^Department of Mathematics, Research Institute for Natural Sciences, Hanyang University, Seoul 133-791, Republic of Korea; ^3^Department of Mathematics Education, Gyeongsang National University, Jinju 660-701, Republic of Korea

## Abstract

The notions of int-soft semigroups and int-soft left (resp., right) ideals are introduced, and several properties are investigated. Using these notions and the notion of inclusive set, characterizations of subsemigroups and left (resp., right) ideals are considered. Using the notion of int-soft products, characterizations of int-soft semigroups and int-soft left (resp., right) ideals are discussed. We prove that the soft intersection of int-soft left (resp., right) ideals (resp., int-soft semigroups) is also int-soft left (resp., right) ideals (resp., int-soft semigroups). The concept of int-soft quasi-ideals is also introduced, and characterization of a regular semigroup is discussed.

## 1. Introduction

Molodtsov [1] introduced the concept of soft set as a new mathematical tool for dealing with uncertainties. He pointed out several directions for the applications of soft set theory. At present, works on soft set theory are progressing rapidly. Maji et al. [2] described the application of soft set theory to a decision making problem. Maji et al. [3] also studied several operations on the theory of soft sets.

Çağman and Enginoğlu [4] introduced fuzzy parameterized (FP) soft sets and their related properties. They proposed a decision making method based on FP-soft set theory and provided an example which shows that the method can be successfully applied to the problems that contain uncertainties. Decision making based on soft sets was further developed in [5–8]. It is worth noting that soft sets are closely related to many other soft computing models such as rough sets and fuzzy sets. Feng and Li [9] initiated soft approximation spaces and soft rough sets, which extended Pawlak's rough sets. Moreover, Feng [10] considered the application of soft rough approximations in multicriteria group decision making problems. Akta*ş* and Çağman [11] studied the basic concepts of soft set theory and compared soft sets to fuzzy and rough sets, providing examples to clarify their differences. They also discussed the notion of soft groups. After that, many algebraic properties of soft sets were studied by several researchers (see [12–26]). Recently, Feng et al. [27] investigated the relationships among five different types of soft subsets. They also explored free soft algebras associated with soft product operations, showing that soft sets have some nonclassical algebraic properties. In this paper, we introduce the notion of int-soft semigroups and int-soft left (resp., right) ideals. Using these notions, we provide characterizations of subsemigroups and left (resp., right) ideals. Using the notion of inclusive set, we also establish characterizations of subsemigroups and left (resp., right) ideals. Using the notion of int-soft products, we give characterizations of int-soft semigroups and int-soft left (resp., right) ideals. We show that the soft intersection of int-soft left (resp., right) ideals (resp., int-soft semigroups) is also int-soft left (resp., right) ideals (resp., int-soft semigroups). We also introduce the concept of int-soft quasi-ideals and discuss a characterization of a regular semigroup by using the notion of int-soft quasi-ideals.

## 2. Preliminaries

Let *S* be a semigroup. Let *A* and *B* be subsets of *S*. Then the multiplication of *A* and *B* is defined as follows:

(1) $$\begin{array}{c} \;\;AB=\left\{ab\in S\;|\;a\in A,b\in B\right\}. \end{array}$$

A semigroup *S* is said to be regular if for every *x* ∈ *S* there exists *a* ∈ *S* such that *xax* = *x*.

A nonempty subset *A* of *S* is called

- a subsemigroup of *S* if *AA*⊆*A*, that is, *ab* ∈ *A* for all *a*, *b* ∈ *A*,
- a left (resp., right) ideal of *S* if *SA*⊆*A* (resp., *SA*⊆*A*), that is, *xa* ∈ *A* (resp., *ax* ∈ *A*) for all *x* ∈ *S* and *a* ∈ *A*,
- a two-sided ideal of *S* if it is both a left and a right ideal of *S*,
- a quasi-ideal of *S* if *AS*∩*SA*⊆*A*.

A soft set theory was introduced by Molodtsov [1], and Çağman and Enginoğlu [5] provided new definitions and various results on soft set theory.

In what follows, let *U* be an initial universe set and let *E* be a set of parameters. Let *P*(*U*) denote the power set of *U* and *A*, *B*, *C*,…⊆*E*.

**Definition 1 (see [1, 5]).**

A soft set (*α*, *A*) over *U* is defined to be the set of ordered pairs

(2) $$\begin{array}{c} \left(\alpha ,A\right):=\left\{\left(x,\alpha \left(x\right)\right):x\in E,\alpha \left(x\right)\in P\left(U\right)\right\}, \end{array}$$

where *α* : *E* → *P*(*U*) such that *α*(*x*) = *∅* if *x* ∉ *A*.

The function *α* is called approximate function of the soft set (*α*, *A*). The subscript *A* in the notation *α* indicates that *α* is the approximate function of (*α*, *A*).

**Definition 2 (see [28]).**

Assume that *E* has a binary operation ↪. For any nonempty subset *A* of *E*, a soft set (*α*, *A*) over *U* is said to be intersectional over *U* if it satisfies

(3) (x↪y∈A⟹α(x)∩α(y)⊆α(x↪y))                  (∀x,y∈A).

For a soft set (*α*, *A*) over *U* and a subset *γ* of *U*, the *γ*-inclusive set of (*α*, *A*), denoted by *i*_*A*_(*α*; *γ*), is defined to be the set

(4) $$\begin{array}{c} \;\;i_{A}\left(\alpha ;\gamma \right):=\left\{x\in A\;|\;\gamma \subseteq \alpha \left(x\right)\right\}. \end{array}$$

## 3. Intersectional Soft Ideals

In what follows, we take *E* = *S*, as a set of parameters, which is a semigroup unless otherwise specified.

**Definition 3 .**

A soft set (*α*, *S*) over *U* is called an intersectional soft semigroup (briefly, int-soft semigroup) over *U* if it satisfies

(5) $$\begin{array}{c} \;\;\left(\forall x,y\in S\right)\;\left(\alpha \left(x\right)\cap \alpha \left(y\right)\subseteq \alpha \left(xy\right)\right). \end{array}$$

**Definition 4 .**

A soft set (*α*, *S*) over *U* is called an intersectional soft left (resp., right) ideal (briefly, int-soft left (resp., right) ideal) over *U* if it satisfies

(6) $$\begin{array}{c} \;\;\left(\forall x,y\in S\right)\;\left(\alpha \left(xy\right)⊇\alpha \left(y\right)\left(\text{resp}.,\alpha \left(xy\right)⊇\alpha \left(x\right)\right)\right). \end{array}$$

If a soft set (*α*, *S*) over *U* is both an int-soft left ideal and an int-soft right ideal over *U*, we say that (*α*, *S*) is an intersectional soft two-sided ideal (briefly, int-soft two-sided ideal) over *U*.

**Example 5 .**

Let *S* = {*a*, *b*, *c*, *d*} be a semigroup with the following Cayley table:

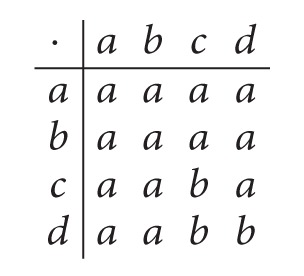
(7)
Let (*α*, *S*) be a soft set over *U* defined as follows:

(8) α:S⟶P(U), x⟼{γ1,if  x=a,γ2,if  x=b,γ4,if  x=c,γ3,if  x=d,

where *γ*_1_, *γ*_2_, *γ*_3_, and *γ*_4_ are subsets of *U* with *γ*_1_⊋*γ*_2_⊋*γ*_3_⊋*γ*_4_. Then (*α*, *S*) is an int-soft two-sided ideal over *U*.

Obviously, every int-soft left (resp., right) ideal over *U* is an int-soft semigroup over *U*. But the converse is not true as seen in the following example.

**Example 6 .**

Let *S* = {0,1, 2,3, 4,5} be a semigroup with the following Cayley table:

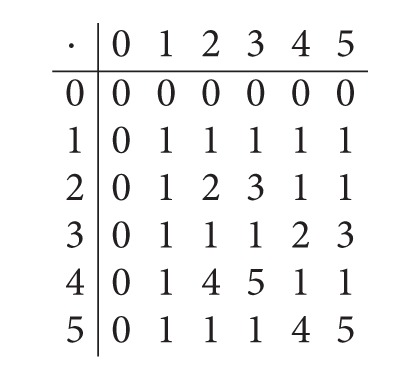
(9)

(1) Let (*α*, *S*) be a soft set over *U* defined as follows:
  (10) α:S⟶P(U), x⟼{γ1,if  x=0,γ2,if  x=1,γ5,if  x∈{2,4},γ4,if  x=3,γ3,if  x=5,
  where *γ*_1_, *γ*_2_, *γ*_3_, *γ*_4_, and *γ*_5_ are subsets of *U* with *γ*_1_⊋*γ*_2_⊋*γ*_3_⊋*γ*_4_⊋*γ*_5_. Then (*α*, *S*) is an int-soft semigroup over *U*. But it is not an int-soft left ideal over *U* since *α*(3 · 5) = *α*(3) = *γ*_4_⊉*γ*_3_ = *α*(5).
(2) Let (*β*, *S*) be a soft set over *U* defined as follows:
  (11) β:S⟶P(U), x⟼{γ1if  x∈{0,1},γ3if  x=2,γ2if  x=3,γ4if  x∈{4,5},
  where *γ*_1_, *γ*_2_, *γ*_3_, and *γ*_4_ are subsets of *U* with *γ*_1_⊋*γ*_2_⊋*γ*_3_⊋*γ*_4_. Then (*β*, *S*) is an int-soft semigroup over *U*. But it is not an int-soft right ideal over *U* since *β*(3 · 4) = *β*(2) = *γ*_3_⊉*γ*_2_ = *β*(3).

For a nonempty subset *A* of *S*, define a map *χ*_*A*_ as follows:

(12) χA:S⟶P(U), x⟼{U,if  x∈  A,∅,otherwise.

Then (*χ*_*A*_, *S*) is a soft set over *U*, which is called the characteristic soft set. The soft set (*χ*_*S*_, *S*) is called the identity soft set over *U*.

**Theorem 7 .**

For any nonempty subset *A* of  *S*, the following are equivalent.

1. *A* is a left (resp., right) ideal of  *S*.
2. The characteristic soft set (*χ*_*A*_, *S*) over  *U* is an int-soft left (resp., right) ideal over  *U*.

**Proof**

Assume that *A* is a left ideal of *S*. For any *x*, *y* ∈ *S*, if *y* ∉ *A* then *χ*_*A*_(*xy*)⊇*∅* = *χ*_*A*_(*y*). If *y* ∈ *A*, then *xy* ∈ *A* since *A* is a left ideal of *S*. Hence *χ*_*A*_(*xy*) = *U* = *χ*_*A*_(*y*). Therefore (*χ*_*A*_, *S*) is an int-soft left ideal over *U*. Similarly, (*χ*_*A*_, *S*) is an int-soft right ideal over *U* when *A* is a right ideal of *S*.

Conversely suppose that (*χ*_*A*_, *S*) is an int-soft left ideal over *U*. Let *x* ∈ *S* and *y* ∈ *A*. Then *χ*_*A*_(*y*) = *U*, and so *χ*_*A*_(*xy*)⊇*χ*_*A*_(*y*) = *U*; that is, *χ*_*A*_(*xy*) = *U*. Thus *xy* ∈ *A* and therefore *A* is a left ideal of *S*. Similarly, we can show that if (*χ*_*A*_, *S*) is an int-soft right ideal over *U*, then *A* is a right ideal of *S*.

**Corollary 8 .**

For any nonempty subset *A* of  *S*, the following are equivalent.

1. *A* is a two-sided ideal of  *S*.
2. The characteristic soft set (*χ*_*A*_, *S*) over  *U*  is an int-soft two-sided ideal over  *U*.

**Theorem 9 .**

A soft set (*α*, *S*) over  *U* is an int-soft semigroup over *U* if and only if the nonempty *γ*-inclusive set of (*α*, *S*) is a subsemigroup of  *S* for all *γ*⊆*U*.

**Proof**

Assume that (*α*, *S*) over *U* is an int-soft semigroup over *U*. Let *γ*⊆*U* be such that *i*_*S*_(*α*; *γ*) ≠ *∅*. Let *x*, *y* ∈ *i*_*S*_(*α*; *γ*). Then *α*(*x*)⊇*γ* and *α*(*y*)⊇*γ*. It follows from (5) that

(13) $$\begin{array}{c} \;\;\alpha \left(xy\right)⊇\alpha \left(x\right)\cap \alpha \left(y\right)⊇\gamma , \end{array}$$

so that *xy* ∈ *i*_*S*_(*α*; *γ*). Thus *i*_*S*_(*α*; *γ*) is a subsemigroup of *S*.

Conversely, suppose that the nonempty *γ*-inclusive set of (*α*, *S*) is a subsemigroup of *S* for all *γ*⊆*U*. Let *x*, *y* ∈ *S* be such that *α*(*x*) = *γ*_*x*_ and *α*(*y*) = *γ*_*y*_. Taking *γ* = *γ*_*x*_∩*γ*_*y*_ implies that *x*, *y* ∈ *i*_*S*_(*α*; *γ*). Hence *xy* ∈ *i*_*S*_(*α*; *γ*), and so *α*(*xy*)⊇*γ* = *γ*_*x*_∩*γ*_*y*_ = *α*(*x*)∩*α*(*y*). Therefore (*α*, *S*) is an int-soft semigroup over *U*.

**Theorem 10 .**

A soft set (*α*, *S*) over *U* is an int-soft left (resp., right) ideal over *U* if and only if the nonempty *γ*-inclusive set of  (*α*, *S*) is a left (resp., right) ideal of  *S* for all *γ*⊆*U*.

**Proof**

It is the same as the proof of Theorem 9.

**Corollary 11 .**

A soft set (*α*, *S*) over  *U* is an int-soft two-sided ideal over *U* if and only if the nonempty *γ*-inclusive set of  (*α*, *S*) is a two-sided ideal of  *S* for all *γ*⊆*U*.

For any soft sets (*α*, *S*) and (*β*, *S*) over *U*, we define

(14) $$\begin{array}{c} \left(\alpha ,S\right)\tilde{\subseteq }\left(\beta ,S\right)\;\text{if}\;\;\alpha \left(x\right)\subseteq \beta \left(x\right)\;\forall x\in S. \end{array}$$

The soft union of (*α*, *S*) and (*β*, *S*) is defined to be the soft set $\left(\alpha \;\tilde{\cup }\;\beta ,S\right)$ over *U* in which $\alpha \;\tilde{\cup }\;\beta$ is defined by

(15) $$\begin{array}{c} \;\;\left(\alpha \tilde{\cup }\beta \right)\left(x\right)=\alpha \left(x\right)\cup \beta \left(x\right)\;\forall x\in S. \end{array}$$

The* soft intersection* of (*α*, *S*) and (*β*, *S*) is defined to be the soft set $\left(\alpha \;\tilde{\cap }\;\beta ,S\right)$ over *U* in which $\alpha \;\tilde{\cap }\;\beta$ is defined by

(16) $$\begin{array}{c} \;\;\left(\alpha \;\;\tilde{\cap }\;\;\beta \right)\left(x\right)=\alpha \left(x\right)\cap \beta \left(x\right)\;\forall x\in S. \end{array}$$

The int-soft product of (*α*, *S*) and (*β*, *S*) is defined to be the soft set $\left(\alpha \;\;\tilde{\circ }\;\;\beta ,S\right)$ over *U* in which $\alpha \;\;\tilde{\circ }\;\;\beta$ is a mapping from *S* to *P*(*U*) given by

(17) (α  ∘~  β)(x) ={⋃x=yz{α(y)∩β(z)},if  ∃y,z∈S  such  that  x=yz,∅,otherwise.  

**Proposition 12 .**

Let  (*α*_1_, *S*), (*α*_2_, *S*), (*β*_1_, *S*), and  (*β*_2_, *S*) be soft sets over *U*. If

(18) $$\begin{array}{c} \left(\alpha_{1},S\right)\tilde{\subseteq }\left(\beta_{1},S\right),\;\;\left(\alpha_{2},S\right)\;\tilde{\subseteq }\;\left(\beta_{2},S\right), \end{array}$$

then $\left(\alpha_{1}\;\;\tilde{\circ }\;\alpha_{2},S\right)\;\tilde{\subseteq }\;\left(\beta_{1}\tilde{\circ }\beta_{2},S\right)$.

**Proof**

Let *x* ∈ *S*. If *x* is not expressed as *x* = *yz* for *y*, *z* ∈ *S*, then clearly

(19) $$\begin{array}{c} \left(\alpha_{1}\;\tilde{\circ }\;\alpha_{2},S\right)\;\tilde{\subseteq }\;\left(\beta_{1}\;\;\tilde{\circ }\;\beta_{2},S\right). \end{array}$$

Suppose that there exist *y*, *z* ∈ *S* such that *x* = *yz*. Then

(20) (α1∘~  α2)(x)=⋃x=yz{α1(y)∩α2(z)}⊆⋃x=yz{β1(y)∩β2(z)}=(β1∘~  β2)(x).

Therefore $\left(\alpha_{1}\tilde{\circ }\;\alpha_{2},S\right)\;\tilde{\subseteq }\;\left(\beta_{1}\tilde{\circ }\;\beta_{2},S\right)$.

**Lemma 13 .**

Let (*χ*_*A*_, *S*) and (*χ*_*B*_, *S*) be characteristic soft sets over  *U* where *A* and *B* are nonempty subsets of  *S*. Then the following properties hold:

1. $\chi_{A}\;\tilde{\cap }\;\chi_{B}=\chi_{A\cap B}$,
2. $\chi_{A}\;\;\tilde{\circ }\;\;\chi_{B}=\chi_{AB}$.

**Proof**

(1) Let *x* ∈ *S*. If *x* ∈ *A*∩*B*, then *x* ∈ *A* and *x* ∈ *B*. Thus we have

(21) $$\begin{array}{c} \left(\chi_{A}\;\;\tilde{\cap }\;\;\chi_{B}\right)\left(x\right)=\chi_{A}\left(x\right)\cap \chi_{B}\left(x\right)=U=\chi_{A\cap B}\left(x\right). \end{array}$$

If *x* ∉ *A*∩*B*, then *x* ∉ *A* or *x* ∉ *B*. Hence we have

(22) $$\begin{array}{c} \left(\chi_{A}\;\;\tilde{\cap }\;\;\chi_{B}\right)\left(x\right)=\chi_{A}\left(x\right)\cap \chi_{B}\left(x\right)=\emptyset =\chi_{A\cap B}\left(x\right). \end{array}$$

Therefore $\chi_{A}\;\tilde{\cap }\;\chi_{B}=\chi_{A\cap B}$.

(2) For any *x* ∈ *S*, suppose *x* ∈ *AB*. Then there exist *a* ∈ *A* and *b* ∈ *B* such that *x* = *ab*. Thus we have

(23) (χA  ∘~  χB)(x)=⋃x=yz{χA(y)∩χB(z)}⊇χA(a)∩χB(b)=U,

and so $\left(\chi_{A}\;\;\tilde{\circ }\;\;\chi_{B}\right)(x)=U$. Since *x* ∈ *AB*, we get *χ*_*AB*_(*x*) = *U*. Suppose *x* ∉ *AB*. Then *x* ≠ *ab* for all *a* ∈ *A* and *b* ∈ *B*. If *x* = *yz* for some *y*, *z* ∈ *S*, then *y* ∉ *A* or *z* ∉ *B*. Hence

(24) $$\begin{array}{c} \left(\chi_{A}\;\;\tilde{\circ }\;\;\chi_{B}\right)\left(x\right)=\underset{x=yz}{⋃}\left\{\chi_{A}\left(y\right)\cap \chi_{B}\left(z\right)\right\}=\emptyset =\chi_{AB}\left(x\right). \end{array}$$

If *x* ≠ *yz* for all *x*, *y* ∈ *S*, then

(25) $$\begin{array}{c} \left(\chi_{A}\;\;\tilde{\circ }\;\;\chi_{B}\right)\left(x\right)=\emptyset =\chi_{AB}\left(x\right). \end{array}$$

In any case, we have $\chi_{A}\;\;\tilde{\circ }\;\;\chi_{B}=\chi_{AB}$.

**Theorem 14 .**

A soft set (*α*, *S*) over *U* is an int-soft semigroup over *U* if and only if $\left(\alpha \;\;\tilde{\circ }\;\;\alpha ,S)\;\tilde{\subseteq }\;(\alpha ,S\right)$.

**Proof**

Assume that $\left(\alpha \;\;\tilde{\circ }\;\;\alpha ,S)\;\tilde{\subseteq }\;(\alpha ,S\right)$ and let *x*, *y* ∈ *S*. Then

(26) $$\begin{array}{c} \;\;\alpha \left(xy\right)⊇\left(\alpha \;\;\tilde{\circ }\;\;\alpha \right)\left(xy\right)⊃\alpha \left(x\right)\cap \alpha \left(y\right), \end{array}$$

and so (*α*, *S*) is an int-soft semigroup over *U*.

Conversely, suppose that (*α*, *S*) is an int-soft semigroup over *U*. Then *α*(*x*)⊇*α*(*y*)∩*α*(*z*) for all *x* ∈ *S* with *x* = *yz*. Thus

(27) $$\begin{array}{c} \;\;\alpha \left(x\right)⊇\underset{x=yz}{⋃}\left\{\alpha \left(y\right)\cap \alpha \left(z\right)\right\}=\left(\alpha \;\;\tilde{\circ }\;\;\alpha \right)\left(x\right) \end{array}$$

for all *x* ∈ *S*. Hence $\left(\alpha \;\;\tilde{\circ }\;\;\alpha ,S)\;\tilde{\subseteq }\;(\alpha ,S\right)$.

**Theorem 15 .**

For the identity soft set (*χ*_*S*_, *S*) and a soft set (*β*, *S*) over  *U*, the following are equivalent:

1. (*β*, *S*) is an int-soft left ideal over *U*,
2. $\left(\chi_{S}\;\;\tilde{\circ }\;\;\beta ,S\right)\;\tilde{\subseteq }\;\left(\beta ,S\right)$.

**Proof**

Suppose that (*β*, *S*) is an int-soft left ideal over *U*. Let *x* ∈ *S*. If *x* = *yz* for some *y*, *z* ∈ *S*, then

(28) (χS  ∘~  β)(x)=⋃x=yz{χS(y)∩β(z)}⊆⋃x=yz{U∩β(yz)}=β(x).

Otherwise, we have $\left(\chi_{S}\;\;\tilde{\circ }\;\;\beta \right)(x)=\emptyset \subseteq \beta (x)$. Therefore $\left(\chi_{S}\;\;\tilde{\circ }\;\;\beta ,S\right)\;\tilde{\subseteq }\;\left(\beta ,S\right)$.

Conversely, assume that $\left(\chi_{S}\;\;\tilde{\circ }\;\;\beta ,S\right)\;\tilde{\subseteq }\;\left(\beta ,S\right)$. For any *x*, *y* ∈ *S*, we have

(29) β(xy)⊇(χS  ∘~  β)(xy)⊇χS(x)∩β(y)=U∩β(y)=β(y).

Hence (*β*, *S*) is an int-soft left ideal over *U*.

Similarly, we have the following theorem.

**Theorem 16 .**

For the identity soft set (*χ*_*S*_, *S*) over *U* and a soft set (*β*, *S*) over *U*, the following assertions are equivalent:

1. (*β*, *S*) is an int-soft right ideal over *U*,
2. $\left(\beta \;\;\tilde{\circ }\;\;\chi_{S},S\right)\;\tilde{\subseteq }\;\left(\beta ,S\right)$.

**Corollary 17 .**

For the identity soft set (*χ*_*S*_, *S*) over *U* and a soft set (*β*, *S*) over *U*, the following assertions are equivalent:

1. (*β*, *S*) is an int-soft two-sided ideal over *U*,
2. $\left(\chi_{S}\;\;\tilde{\circ }\;\;\beta ,S\right)\;\tilde{\subseteq }\;\left(\beta ,S\right)$ and $\left(\beta \;\;\tilde{\circ }\;\;\chi_{S},S\right)\;\tilde{\subseteq }\;\left(\beta ,S\right)$.

**Theorem 18 .**

If (*α*, *S*) and (*β*, *S*) are int-soft semigroups over  *U*, then so is the soft intersection $\left(\alpha \;\;\tilde{\cap }\;\;\beta ,S\right)$.

**Proof**

Let *x*, *y* ∈ *S*. Then

(30) (α  ∩~  β)(xy)=α(xy)∩β(xy)⊇(α(x)∩α(y))∩(β(x)∩β(y))    =(α(x)∩β(x))∩(α(y)∩β(y))  =(α  ∩~  β)(x)∩(α  ∩~  β)(y).

Thus $\left(\alpha \;\;\tilde{\cap }\;\;\beta ,S\right)$ is an int-soft semigroup over *U*.

By similar manner, we can prove the following theorem.

**Theorem 19 .**

If  (*α*, *S*) and (*β*, *S*) are int-soft left ideals (resp., int-soft right ideals) over *U*, then so is the soft intersection $\left(\alpha \;\;\tilde{\cap }\;\;\beta ,S\right)$.

**Corollary 20 .**

If  (*α*, *S*) and (*β*, *S*) are int-soft two-sided ideals over *U*, then so is the soft intersection $\left(\alpha \;\;\tilde{\cap }\;\;\beta ,S\right)$.

**Theorem 21 .**

Let  (*α*, *S*) and (*β*, *S*) be soft sets over *U*. If (*α*, *S*) is an int-soft left ideal over *U*, then so is the int-soft product $\left(\alpha \;\;\tilde{\circ }\;\;\beta ,S\right)$.

**Proof**

Let  *x*, *y* ∈ *S*. If *y* = *ab* for some *a*, *b* ∈ *S*, then *xy* = *x*(*ab*) = (*xa*)*b* and

(31) (α  ∘~  β)(y)=⋃y=ab{α(a)∩β(b)}⊆⋃xy=(xa)b{α(xa)∩β(b)}⊆⋃xy=cb{α(c)∩β(b)}=(α  ∘~  β)(xy).

If *y* is not expressible as *y* = *ab* for *a*, *b* ∈ *S*, then $\left(\alpha \;\;\tilde{\circ }\;\;\beta \right)(y)=\emptyset \subseteq \left(\alpha \;\;\tilde{\circ }\;\;\beta \right)(xy)$. Thus $\left(\alpha \;\;\tilde{\circ }\;\;\beta \right)(y)\subseteq \left(\alpha \;\;\tilde{\circ }\;\;\beta \right)(xy)$ for all *x*, *y* ∈ *S*, and so $\left(\alpha \;\tilde{\circ }\;\beta ,S\right)$ is an int-soft left ideal over *U*.

Similarly, we have the following theorem.

**Theorem 22 .**

Let (*α*, *S*) and  (*β*, *S*) be soft sets over *U*. If (*β*, *S*) is an int-soft right ideal over *U*, then so is the int-soft product $\left(\alpha \;\;\tilde{\circ }\;\;\beta ,S\right)$.

**Corollary 23 .**

The int-soft product of two int-soft two-sided ideals over *U* is an int-soft two-sided ideal over *U*.

Let (*α*, *S*) be a soft set over *U*. For a subset *γ* of *U* with *i*_*S*_(*α*; *γ*) ≠ *∅*, define a soft set (α*, *S*) over *U* by

(32) α∗:S⟶P(U), x⟼{α(x),if  x∈iS(α;γ),δ,otherwise,

where *δ* is a subset of *U* with *δ*⊊*α*(*x*).

**Theorem 24 .**

If  (*α*, *S*) is an int-soft semigroup over *U*, then so is (α*, *S*).

**Proof**

Let *x*, *y* ∈ *S*. If  *x*, *y* ∈ *i*_*S*_(*α*; *γ*), then *xy* ∈ *i*_*S*_(*α*; *γ*) since *i*_*S*_(*α*; *γ*) is a subsemigroup of *S* by Theorem 9. Hence we have

(33) $$\begin{array}{c} \;\;\alpha^{*}\left(xy\right)=\alpha \left(xy\right)⊇\alpha \left(x\right)\cap \alpha \left(y\right)=\alpha^{*}\left(x\right)\cap \alpha^{*}\left(y\right). \end{array}$$

If  *x* ∉ *i*_*S*_(*α*; *γ*) or *y* ∉ *i*_*S*_(*α*; *γ*), then α*(*x*) = *δ* or α*(*y*) = *δ*. Thus

(34) $$\begin{array}{c} \;\;\alpha^{*}\left(xy\right)⊇\delta =\alpha^{*}\left(x\right)\cap \alpha^{*}\left(y\right). \end{array}$$

Therefore (α*, *S*) is an int-soft semigroup over *U*.

By similar manner, we can prove the following theorem.

**Theorem 25 .**

If  (*α*, *S*) is an int-soft left ideal (resp., int-soft right ideal) over *U*, then so is (α*, *S*).

**Corollary 26 .**

If  (*α*, *S*) is an int-soft two-sided ideal over *U*, then so is (α*, *S*).

**Theorem 27 .**

If  (*α*, *S*) is an int-soft right ideal over *U* and (*β*, *S*) is an int-soft left ideal over *U*, then $\left(\alpha \;\;\tilde{\circ }\;\;\beta ,S\right)\;\tilde{\subseteq }\;\left(\alpha \;\tilde{\cap }\;\beta ,S\right)$.

**Proof**

Let *x* ∈ *S*. If *x* is not expressible as *x* = *ab* for *a*, *b* ∈ *S*, then $\left(\alpha \;\;\tilde{\circ }\;\;\beta \right)(x)=\emptyset \subseteq \left(\alpha \;\tilde{\cap }\;\beta \right)(x)$. Assume that there exist *a*, *b* ∈ *S* such that *x* = *ab*. Then

(35) (α  ∘~  β)(x)=⋃x=ab{α(a)∩β(b)}⊆⋃x=ab{α(ab)∩β(ab)}=α(x)∩β(x)=(α  ∩~  β)(x).

In any case, we have $\left(\alpha \;\tilde{\circ }\;\beta ,S\right)\;\tilde{\subseteq }\;\left(\alpha \;\tilde{\cap }\;\beta ,S\right)$.

If we strengthen the condition of the semigroup *S*, then we can induce the reverse inclusion in Theorem 27 as follows.

**Theorem 28 .**

Let *S* be a regular semigroup. If  (*α*, *S*) is an int-soft right ideal over  *U*, then $\left(\alpha \;\tilde{\cap }\;\beta ,S\right)\;\tilde{\subseteq }\;\left(\alpha \;\;\tilde{\circ }\;\;\beta ,S\right)$ for every soft set (*β*, *S*) over  *U*.

**Proof**

Let *x* ∈ *S*. Then there exists *a* ∈ *S* such that *xax* = *x* since *S* is regular. Thus

(36) $$\begin{array}{c} \;\;\left(\alpha \;\;\tilde{\circ }\;\;\beta \right)\left(x\right)=\underset{x=yz}{⋃}\left\{\alpha \left(y\right)\cap \beta \left(z\right)\right\}. \end{array}$$

On the other hand, we have

(37) $$\begin{array}{c} \;\;\left(\alpha \;\;\tilde{\cap }\;\;\beta \right)\left(x\right)=\alpha \left(x\right)\cap \beta \left(x\right)\subseteq \alpha \left(xa\right)\cap \beta \left(x\right) \end{array}$$

since (*α*, *S*) is an int-soft right ideal over *U*. Since *xax* = *x*, we obtain

(38) $$\begin{array}{c} \;\;\alpha \left(xa\right)\cap \beta \left(x\right)\subseteq \underset{x=yz}{⋃}\left\{\alpha \left(y\right)\cap \beta \left(z\right)\right\}=\left(\alpha \;\;\tilde{\circ }\;\;\beta \right)\left(x\right). \end{array}$$

Therefore $\left(\alpha \;\;\tilde{\cap }\;\;\beta \right)(x)\subseteq \left(\alpha \;\;\tilde{\circ }\;\;\beta \right)(x)$, and so $\left(\alpha \;\;\tilde{\cap }\;\;\beta ,S\right)\;\;\tilde{\subseteq }\;\;\left(\alpha \;\;\tilde{\circ }\;\;\beta ,S\right)$.

In a similar way we prove the following.

**Theorem 29 .**

Let *S* be a regular semigroup. If  (*β*, *S*) is an int-soft left ideal over  *U*, then $\left(\alpha \;\;\tilde{\cap }\;\;\beta ,S\right)\;\tilde{\subseteq }\;\left(\alpha \;\;\tilde{\circ }\;\;\beta ,S\right)$ for every soft set (*α*, *S*) over  *U*.

**Theorem 30 .**

If a semigroup *S* is regular, then $\left(\alpha \;\tilde{\cap }\;\beta ,S\right)=\left(\alpha \;\;\tilde{\circ }\;\;\beta ,S\right)$ for every int-soft right ideal (*α*, *S*) and int-soft left ideal (*β*, *S*) over  *U*.

**Proof**

Assume that *S* is a regular semigroup and let (*α*, *S*) and (*β*, *S*) be an int-soft right ideal and an int-soft left ideal, respectively, over *U*. By Theorem 28, we have $\left(\alpha \;\tilde{\cap }\;\beta ,S\right)\;\tilde{\subseteq }\;\left(\alpha \;\tilde{\circ }\;\beta ,S\right)$. Since $\left(\alpha \;\tilde{\circ }\;\beta ,S\right)\;\tilde{\subseteq }\;\left(\alpha \;\tilde{\cap }\;\beta ,S\right)$ by Theorem 27, we have $\left(\alpha \;\tilde{\cap }\;\beta ,S\right)=\left(\alpha \;\tilde{\circ }\;\beta ,S\right)$.

**Definition 31 .**

A soft set (*α*, *S*) over *U* is called an int-soft quasi-ideal over *U* if

(39) $$\begin{array}{c} \;\;\left(\alpha \;\;\tilde{\circ }\;\;\chi_{S},S\right)\tilde{\cap }\left(\chi_{S}\;\;\tilde{\circ }\;\alpha ,S\right)\tilde{\subseteq }\left(\alpha ,S\right). \end{array}$$

Obviously, every int-soft left (resp., right) ideal is an int-soft quasi-ideal over *U*, but the converse does not hold in general.

In fact, we have the following example.

**Example 32 .**

Let *S* = {0, *a*, *b*, *c*} be a semigroup with the following Cayley table:

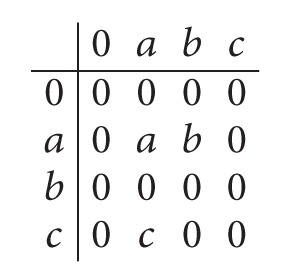
(40)
Let (*α*, *S*) be a soft set over *U* defined as follows:

(41) α:S⟶P(U), x⟼{γ,if  x∈{0,a},∅,if  x∈{b,c},

where *γ* is a subset of *U*. Then (*α*, *S*) is an int-soft quasi-ideal over *U* and is not an int-soft left (resp., right) ideal over *U*.

**Theorem 33 .**

Let *G* be a nonempty subset of  *S*. Then *G* is a quasi-ideal of *S* if and only if the characteristic soft set (*χ*_*G*_, *S*) is an int-soft quasi-ideal over  *U*.

**Proof**

We first assume that *G* is a quasi-ideal of *S*. Let *a* be any element of *S*. If *a* ∈ *G*, then

(42) $$\begin{array}{c} \;\;\left(\left(\chi_{G}\;\;\tilde{\circ }\;\;\chi_{S}\right)\tilde{\cap }\left(\chi_{S}\;\;\tilde{\circ }\;\;\chi_{G}\right)\right)\left(a\right)\tilde{\subseteq }\;\;U=\chi_{G}\left(a\right). \end{array}$$

If *a* ∉ *G*, then *χ*_*G*_(*a*) = *∅*. On the other hand, assume that

(43) $$\begin{array}{c} \;\;\left(\left(\chi_{G}\;\;\tilde{\circ }\;\;\chi_{S}\right)\tilde{\cap }\left(\chi_{S}\;\;\tilde{\circ }\;\;\chi_{G}\right)\right)\left(a\right)=U. \end{array}$$

Then

(44) $$\begin{array}{c} \;\;\underset{a=xy}{⋃}\left\{\chi_{G}\left(x\right)\cap \chi_{S}\left(y\right)\right\}=\left(\chi_{G}\;\;\tilde{\circ }\;\;\chi_{S}\right)\left(a\right)=U,\\ \;\;\underset{a=xy}{⋃}\left\{\chi_{S}\left(x\right)\cap \chi_{G}\left(y\right)\right\}=\left(\chi_{S}\;\;\tilde{\circ }\;\;\chi_{G}\right)\left(a\right)=U. \end{array}$$

This implies that there exist elements *b*, *c*, *d*, and *e* of *S* with *a* = *bc* = *de* such that *χ*_*G*_(*b*) = *U* and *χ*_*G*_(*e*) = *U*. Hence *a* = *bc* = *de* ∈ *GS*∩*SG*⊆*G*, which contradicts that *a* ∉ *G*. Thus we have $\left(\chi_{G}\;\;\tilde{\circ }\;\;\chi_{S},S\right)\;\tilde{\cap }\;\left(\chi_{S}\;\;\tilde{\circ }\;\;\chi_{G},S\right)\;\tilde{\subseteq }\;\left(\chi_{G},S\right)$ and so (*χ*_*G*_, *S*) is an int-soft quasi-ideal over *U*.

Conversely, suppose that (*χ*_*G*_, *S*) is an int-soft quasi-ideal over *U*. Let *a* be any element of *GS*∩*SG*. Then *bx* = *a* = *yc* for some *b*, *c* ∈ *G* and *x*, *y* ∈ *S*. It follows from (39) that

(45) χG(a)⊇((χG  ∘~  χS)∩~(χS  ∘~  χG))(a)=(χG  ∘~  χS)(a)∩(χS  ∘~  χG)(a)=(⋃a=uv{χG(u)∩χS(v)})∩(⋃a=uv{χS(u)∩χG(v)})=(⋃a=uv{χG(u)})∩(⋃a=uv{χG(v)})=U

and so *a* ∈ *G*. Thus *GS*∩*SG*⊆*G*, and hence *G* is a quasi-ideal of *S*.

**Theorem 34 .**

For a semigroup *S*, the following are equivalent:

1. *S* is regular,
2. $\left(\alpha ,S)=(\alpha \;\;\tilde{\circ }\;\;\chi_{S}\;\;\tilde{\circ }\;\;\alpha ,S\right)$ for every int-soft quasi-ideal (*α*, *S*) over  *U*.

**Proof**

Assume that *S* is regular and let *a* ∈ *S*. Then *a* = *a* *xa* for some *x* ∈ *S*. Hence

(46) (α  ∘~  χS  ∘~  α)(a)=⋃a=uv{(α  ∘~  χS)(u)∩α(v)}⊇(α  ∘~  χS)(ax)∩α(a)=(⋃ax=cd{α(c)∩χS(d)})∩α(a)=(⋃ax=cd{α(c)})∩α(a)=α(a),

and so $(\alpha ,S)\;\tilde{\subseteq }\;\left(\alpha \;\;\tilde{\circ }\;\;\chi_{S}\;\;\tilde{\circ }\;\;\alpha ,S\right)$. On the other hand, since (*α*, *S*) is an int-soft quasi-ideal over *U*,

(47) $$\begin{array}{c} \;\;\left(\alpha \;\;\tilde{\circ }\;\;\chi_{S}\;\;\tilde{\circ }\;\;\alpha ,S\right)\tilde{\subseteq }\left(\alpha \;\;\tilde{\circ }\;\;\chi_{S},S\right)\tilde{\cap }\left(\chi_{S}\;\;\tilde{\circ }\;\;\alpha ,S\right)\tilde{\subseteq }\left(\alpha ,S\right). \end{array}$$

Hence $\left(\alpha ,S)=(\alpha \;\;\tilde{\circ }\;\;\chi_{S}\;\;\tilde{\circ }\;\;\alpha ,S\right)$.

Conversely, suppose that (2) is valid and let *A* be a quasi-ideal of *S*. Then *A* *SA*⊆*AS*∩*SA*⊆*A* and (*χ*_*A*_, *S*) is an int-soft quasi-ideal over *U*. For any *a* ∈ *A*, we have

(48) ⋃a=yz{(χA  ∘~  χS)(y)∩χA(z)}  =((χA  ∘~  χS)  ∘~  χA)(a)=χA(a)=U.

This implies that there exist *b*, *c* ∈ *S* such that $a=bc,\left(\chi_{A}\;\;\tilde{\circ }\;\;\chi_{S}\right)(b)=U$ and *χ*_*A*_(*c*) = *U*. Then

(49) $$\begin{array}{c} \;\;U=\left(\chi_{A}\;\;\tilde{\circ }\;\;\chi_{S}\right)\left(b\right)=\underset{b=pq}{⋃}\left\{\chi_{A}\left(p\right)\cap \chi_{S}\left(q\right)\right\}, \end{array}$$

and so *b* = *st*,  *χ*_*A*_(*s*) = *U* = *χ*_*S*_(*t*) for some *s*, *t* ∈ *S*. It follows that *c*, *s* ∈ *A* and *t* ∈ *S* so that *a* = *bc* = (*st*)*c* ∈ *A* *SA*. Hence *A*⊆*A* *SA*, and thus *A* = *A* *SA*. Therefore *S* is regular.
